# Life Cycle Replacement by Gene Introduction under an Allee Effect in Periodical Cicadas

**DOI:** 10.1371/journal.pone.0018347

**Published:** 2011-04-06

**Authors:** Yukiko Nariai, Saki Hayashi, Satoru Morita, Yoshitaka Umemura, Kei-ichi Tainaka, Teiji Sota, John R. Cooley, Jin Yoshimura

**Affiliations:** 1 Department of Systems Engineering, Shizuoka University, Hamamatsu, Japan; 2 Faculty of Information Science, Shizuoka University, Hamamatsu, Japan; 3 Department of Zoology, Kyoto University, Kyoto, Japan; 4 Department of Ecology and Evolutionary Biology, University of Connecticut, Storrs, Connecticut, United States of America; 5 Marine Biosystems Research Center, Chiba University, Kamogawa, Chiba, Japan; 6 Department of Environmental and Forest Biology, State University of New York College of Environmental Science and Forestry, Syracuse, New York, United States of America; University of Maribor, Slovenia

## Abstract

Periodical cicadas (*Magicicada* spp.) in the USA are divided into three species groups (-decim, -cassini, -decula) of similar but distinct morphology and behavior. Each group contains at least one species with a 17-year life cycle and one with a 13-year cycle; each species is most closely related to one with the other cycle. One explanation for the apparent polyphyly of 13- and 17-year life cycles is that populations switch between the two cycles. Using a numerical model, we test the general feasibility of life cycle switching by the introduction of alleles for one cycle into populations of the other cycle. Our results suggest that fitness reductions at low population densities of mating individuals (the Allee effect) could play a role in life cycle switching. In our model, if the 13-year cycle is genetically dominant, a 17-year cycle population will switch to a 13-year cycle given the introduction of a few 13-year cycle alleles under a moderate Allee effect. We also show that under a weak Allee effect, different year-classes (“broods”) with 17-year life cycles can be generated. Remarkably, the outcomes of our models depend only on the dominance relationships of the cycle alleles, irrespective of any fitness advantages.

## Introduction

Cicadas are remarkable singing insects in tropical and temperate forests that belongs to family Cicadidae (Suborder: Homoptera; Order: Heteroptera) [1], [2]. Male cicadas sing mating calls, while females are attracted to male calls [3], [4]. Recently females are found to respond to males by wing flicking and mating proceeds with male-female communications [5]. Cicadas are also unique in their long juvenile stages in soil spreading 3–10 years and very short adult lives (a couple weeks), due to their feeding on poor-nutrient xylem water in tree roots [1], [2], [6]. In a short adult stage, females mate with males and lay eggs on small twigs, from where nymphs hatch soon or later and drop to the ground, and dig into the soil, where they feed on plant roots [7].

Periodical cicadas (*Magicicada* spp.) in the USA are unusual with excessively long prime-numbered life cycles of 13- or 17-years [8]–[12], [6]. Among all cicadas, they are the only known group with periodicity [cite]. The known maturation determinant of all other cicadas is not time, but cumulative temperature [cite]. Periocical cicadas are also unique in their life histories, characterized by mass, synchronized emergences of millions per acre [13] and are divided into regional populations sharing emergence years (“year-classes,” specifically called “broods”). Three taxonomic groups (-decim, -cassini, and -decula) contain 7 species. The -cassini and -decula groups contain two species: 17-year *M. cassini* and 13-year *M. tredecassini* and 17-year *M. septendecula* and 13-year *M. tredecula*, respectively. The -decim group consists of three species: 17-year *M. septendecim* and 13-year *M. tredecim* and *M. neotredecim*. Each species is most closely related to one with the other life cycle in its own species group [11], [14], and permanent life cycle shifts have been proposed to explain these relationships [11], [14]–[16].

The evolutionary origin of *M. neotredecim* appears to be a permanent life cycle shift from a 17-year to a13-year cycle. Genetic, behavioral, and biogeographic evidence suggest that *M. neotredecim* originated recently from the 17-year species *M. septendecim* [14]–[17]. *M. neotredecim* is indistinguishable from 17-year *M. septendecim* genetically and morphologically, and it shows a striking pattern of reproductive character displacement in calling song pitch with the closely related species *M. tredecim* [14], [16].

Within 17-year periodical cicadas, brood formation appears to occur via temporary life cycle shifts, in which large numbers of cicadas emerge off-cycle, perhaps in response to climate fluctuations (“brood shifting”) [11], [18]. Among 17-year broods of periodical cicadas, differences of ±1 or ±4 years appear to be especially common [19]–[23]. Permanent life cycle switching involving small numbers of cicadas is difficult to explain, because small numbers of periodical cicadas may fail to reproduce [24] or be quickly destroyed by predators since *Magicicada* rely on predator satiation by extreme abundance [11], [19], [25]–[27]. Thus, any explanation for life cycle switching in periodical cicadas must take into account Allee Effects acting against small populations or minority life cycle phenotypes [28].

Here we investigate the possibility that life cycle switching between 13- and 17-year cycles can be explained by the introduction of a few life cycle alleles (individuals) into an isolated population with the other cycle. We construct a simple numerical model of hybridization between 17-year and 13-year cycles. Although hybridization and introgression in hybrid zones [29]–[31] have been proposed as one factor stimulating permanent life cycle change in periodical cicadas [32]–[35], our model of gene introduction is not “genetic introgression” in the strict sense, because there are no hybrid zones involved.

In our model life cycle is assumed to be controlled by alleles at a locus under simple Mendelian inheritance, with one cycle (either 17- or 13-year) dominant to the other. Neither allele has a selective advantage except the advantage inherent in a shorter life cycle (generation time). We also consider diploid and haploid (mitochondrial) genes unlinked to the cycle locus. We analyze the evolutionary fates of “cycle alleles” in mixed populations, where varying degrees of an Allee effect [28], [36] are imposed.

## Methods

### Model

**Figure 1:**
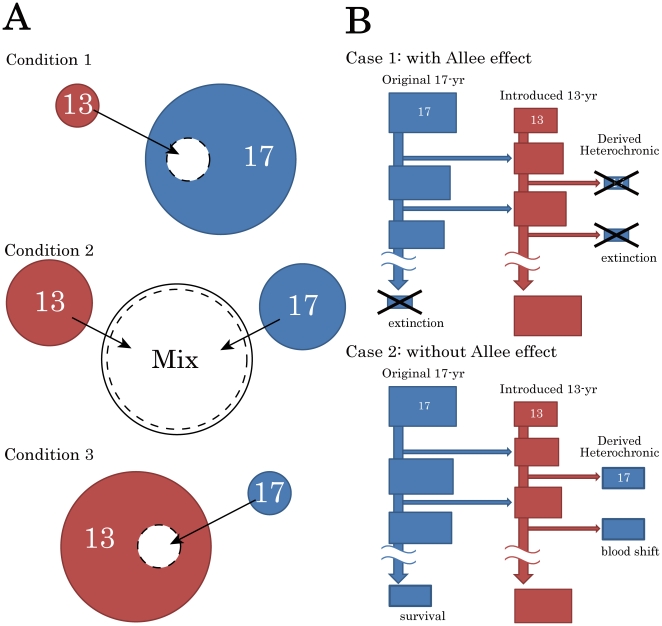
Schematic diagram of life cycle shifting by gene introduction in periodical cicadas. (A) The proportion of 13 (17)-year cycle in the initial mixed populations. Condition 1: a few 13-year individuals are introduced into a large 17-year population (*P*_13_ = 0.1). Condition 2: a 13-year population is mixed with a 17-year population of the same size (*P*_13_ = 0.5). Condition 3: a few 17-year individuals are introduced into a large 13-year population (*P*_13_ = 0.9). (B) Repeated hybridization over time for Condition 1. When few individuals of a 13-year brood are introduced into the 17-year brood (the original population), a small population of heterochronic 17-year broods is produced via 13-year hybrids. Case 1: with an Allee effect. The original and derived 17-year broods are all eliminated by an Allee effect (cross signs indicate extinction). Only the 13-year broods survive in the end. Case 2: without an Allee effect. In 17-year cycles brood shifts may result in all possible 17 broods. All the original and derived 17-year broods may survive in the end.

Our numerical model starts with various proportions of two pure populations with alleles for either 13- or 17-year life cycles (Figure 1). In the beginning of each simulation, the two populations are mixed (Figure 1A), and the proportion of 13(17)-year individuals is varied from 0 to 1 (1 to 0). The model keeps track of the population sizes (*N*) of all broods/hybrids as a real number including birth year with juvenile (*N_l,t_*) and adult stages (*N_A,t_*).

Allee effects are set as critical population sizes (extinction thresholds), *N_c_*, below which the population immediately becomes extinct. To test the sensitivity of the model to Allee effects, we varied the extinction thresholds, such that *N_c_* = 0 (control), 1, …, 100. We also varied the initial population sizes, *N_INI_*, ( = 0,…,10,000) and the initial proportions of 13-year cycles, *P*_13_, ( = 0.00,…,1.00).

Rates of hybridization (mating) among the populations/genotypes are assumed to be exactly proportional to the relative population sizes of co-emerging individuals; e.g., no mate choice occurs. The population growth rates (r) of all genotypes are set slightly positive (r = 1.001 unless specified) to counteract population decreases due to the extinction of small (<*N_c_*) populations by an Allee effect (Figure 1B). All simulations run at least 10,000 years, when the proportions of every cycles/broods stabilize.

### Simulation procedures

The model keeps track of the population sizes *N* of heterozygotes and both homozygotes, their emergence schedules (broods), and their growth stages. Population size is calculated as a real number (double precision). Per capita population growth rate, *r*, includes the number of eggs, juvenile survival and adult emergence success in the next generation. Here *r* = 1.001 (slightly positive), to compensate for negative Allee effects. We count the population sizes of females, assuming the numbers of males are the same. We assume that the genetic system of the cicadas follows Mendelian inheritance. The cycle genotypes are a single locus with two alleles: 17-year and 13-year cycle alleles. Other loci are not linked with the cycle loci. We also assume that mitochondrial loci are transmitted exclusively through matrilines.

The genotype of each individual is represented as [(i,j),(k,l)] where i, j, k and l denote genes and the first [second] bracket indicates cycle [non-cycle] genotypes. The population of [(i,j),(k,l)] is denoted as *N* [(i,j),(k,l)]. We assume shorter cycle (13-year) dominance, but we also test longer cycle (17-year) dominance. We denote that *N_A,t_* is the population size of adults emerging at *t* (year) and *N_l,t_*, the population sizes of juveniles yielded (eggs laid) at *t*. The offspring (juvenile) brood size between *N* [(i,j),(k,l)] and *N* [(m,n),(o,p)] is: 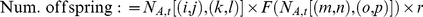
(1)where *F* is the frequency of the target brood, such that
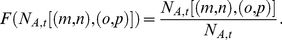
(2)The genotypes of hybrid offspring between cycle genotypes [*i,j*] and [*m,n*] include four possibilities: [*i,m*], [*i,n*], [*j,m*] and [*j,n*]. Thus, the offspring brood size of each genotype is the sum of eq. [1]. For example, the offspring brood size of genotype [(i,m),(k,o)] follows:
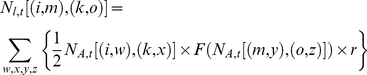
(3)The mitochondrial haplotypes are handled in a similar fashion.

## Results

**Figure 2:**
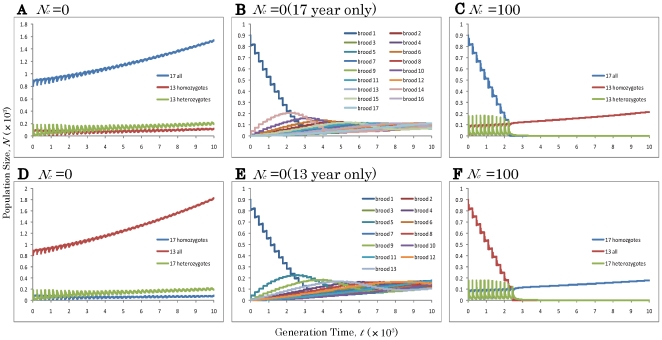
Temporal dynamics of hybridization between 17- and 13-year cycles in periodical cicadas. (A–C): 13-year allele is dominant and (D–F): 17-year is dominant. (A) Without an Allee effect (*N_c_* = 0). (B) The same as (A) for all seventeen 17-yr broods. (C) With an Allee effect (*N_c_* = 100). (D) Without an Allee effect (*N_c_* = 0). (E) The same as (D) for all thirteen 13-yr broods. (F) With an Allee effect (*N_c_* = 100). Other parameters are *N_INI_* = 1000, *P*_13_ = 0.1, and *r* = 1.001.

**Figure 3:**
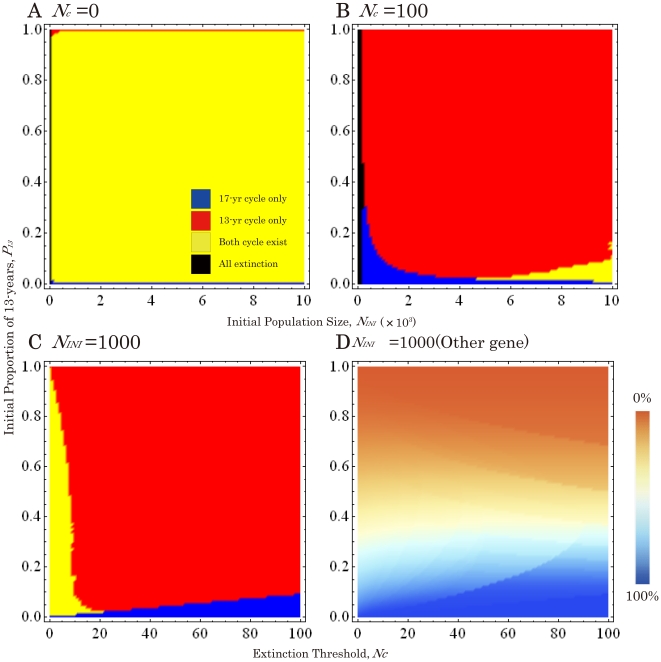
Phase planes of emergent cycles between 17- and 13-year cycles when 13-year gene is dominant. (A) Without an Allee effect (*N_c_* = 0). (B) With an Allee effect (*N_c_* = 100). (C) Under varied Allee effects (*N_c_* = 0∼100) and a constant initial population size (*N_INI_* = 1,000). (D) Phase plane of non-cycle genes. The percent genes originated from 17(13)-year broods are shown in Blue (Orange). Other parameters are *r* = 1.001 and *t* = 10,000.

**Figure 4:**
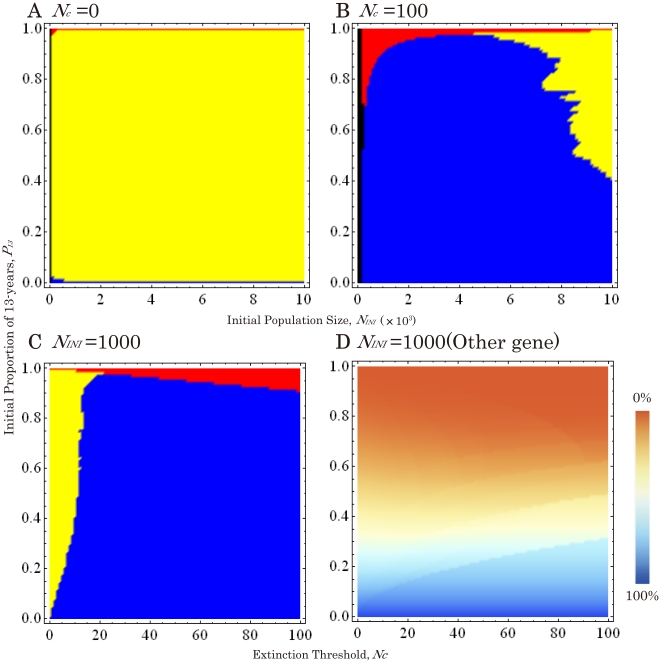
Phase planes of emergent cycles between 17- and 13-year cycles when 17-year gene is dominant. (A) Without an Allee effect (*N_c_* = 0). (B) With an Allee effect (*N_c_* = 100). (C) Under varied Allee effects (*N_c_* = 0∼100) and a constant initial population size (*N_INI_* = 1,000). (D) Phase plane of non-cycle genes. The percent genes originated from 17(13)-year broods are shown in Blue (Orange). Other parameters are *r* = 1.001 and *t* = 10,000.

The simulation results for both 13- and 17-year dominance are shown in Figures 2, 3 and 4. These two outcomes are almost mirror images with each other (Figures 3 and 4).

In the absence of Allee effects, the hybrid population predominantly exhibits the genetically dominant cycle (13-year in Figure 2A; 17-year in Figure 2D). At the same time, offspring with homozygous recessive cycle alleles sequentially form new 4-year offset broods (4-year advanced 17-year broods in Figure 2B; 4-year delayed 13-year broods in Figure 2E). This results in existence of all possible broods of the recessive cycles with almost equal frequency after 10,000 years (all seventeen 17-year broods in Figure 2B; all thirteen 13-year broods in Figure 2E).

In contrast, when moderate to strong levels of Allee effects are present (*N_c_* = 100), a population of a recessive cycle may shift entirely to the genetically dominant cycle after the introduction of a few individuals with the dominant alleles (*P*_13_≤0.10 in Figure 2C and *P*_13_≥0.90 in Figure 2F). Under these conditions, all broods of the recessive cycle disappear (Figure 1B).

When the proportions of 13-year cycle individuals in a mixed population, *P*_13_, are varied (*P*_13_ = 0.0∼1.0; Figures 3 and 4), both cycles survive (Figures 3A and 4A) when no Allee effect is applied (*N_c_* = 0). When an Allee effect (*N_c_*≥100) is applied, however, the alleles for a recessive cycle are replaced by those for a dominant cycle under wide ranges of initial conditions (Figures 3B and 4B). When the initial population size is relatively small (*N_INI_* = ca.1,000∼3,000), the cycle allele replacement occurs even if the initial proportion of the recessive cycle is more than 90% (*P*_13_≤0.1 in Figures 3B and *P*_13_≥0.9 in 4B).

When the initial population size (*N_INI_*) is relatively small (1,000), replacement by a dominant cycle does not happen, instead: brood shifts (the formation of different year classes of the same life cycle) occurs in the recessive cycle if *N_c_* is significantly low (<10 individuals; Figures 2B, 3C, 2E and 4C). The critical thresholds for replacement by the dominant cycle appear to be about 1% of the initial population size (Figures 3C and 4C). Thus brood shifts occur when an Allee effect is weak or absent, and life cycle replacement occurs when it is stronger. The conditions under which the cycle replacements or brood shifts occur appear to depend on the combination of all three parameters: The ratio of critical population size (*N_c_*) to initial population size (*N_INI_*), and the initial proportion (*P*_13_).

We also test the effects of hybridization on loci not linked to the cycle loci (Figures 3D and 4D). The frequencies of such “non-cycle” loci remain unchanged, irrespective of brood formation or life cycle replacement. For example, when the initial proportion of 13-year individuals (*P*_13_) is 0.05, the ending proportion of non-cycle loci originated from 17-year individuals is almost 95%, even if 17-year cycle alleles are replaced completely by 13-year alleles. The results are almost identical for genes with uniparental inheritance such as mitochondrial genes. Thus the proportions of non-cycle loci are essentially determined by the initial proportions of individuals and are unaffected by gene introduction (Figures 3 and 4). All the outcomes are determined quickly, in less than 1,000 generations.

## Discussion

Our model makes an intriguing prediction. Life cycle switching by gene introduction appears to be possible under a moderate level of Allee effects. Surprisingly the direction of switching depends only on genetic dominance: switching from recessive to dominant cycles. That is, (1) from 17-year to 13-year when 13-year is dominant (Figures 2A–C and 3), and (2) from 13-year to 17-year when 17-year is dominant (Figures 2D–F and 4). Given the genetic evidence that 13-year *M. neotredecim* originated from a 17-year ancestral *M. septendecim*, the mechanism postulated here would also suggest that a 13-year cycle allele is dominant. Unfortunately the dominance relationships of life cycle alleles remain unknown in periodical cicadas.

Another unexpected prediction is brood formation in recessive cycles. Under weak or no Allee effects, life cycle switching does not occur; instead brood formations occur in recessive cycles. All possible seventeen 17-year broods may be derived if the 13-year cycle is dominant (Figures 2A–C and 3), while all thirteen 13-year broods are derived if 17-year is dominant (Figures 2D–F and 4). Under this mechanism, many different broods of the recessive cycle can be formed repeatedly by the immigration of individuals bearing dominant alleles into the populations of recessive alleles. These recessive cycles could persist in proximity to a few large broods of the dominant cycle. Given that 12 extant 17-year cicada broods and three extant 13-year cicadas currently exist, 13-year dominance is again consistent with our model.

Our model relies on several assumptions. The current model assumes no mating preferences. The scenario described in the model is effectively one in which a rare-species individual is confronted with the choice of mating with a heterospecific, or being unlikely to mate at all. In several studies of insects and other animals [37]–[41], mating preferences appear to relax under conditions of low conspecific population density. Exactly what periodical cicadas do in such situations remains unknown. In the present periodical cicadas, the -decim group show strikingly different male song pitches and female mating preferences for conspecifics between *M. septendecim* and *M. tredecim* [5], [42] and between *M. neotredecim* and *M. tredecim* even outside of their contact zone [14]. The addition of mating preferences is expected to change the model outcome quantitatively, but the applicability and/or generality of the model may increase otherwise [28].

Second, we assume that neither cycle alleles have a selective disadvantage other than shorter cycle length of 13-year cicadas. The net reproductive rate/generation is kept identical for both the cycles. Importantly, although the 4 year difference in generation time exists, the life cycle evolution is not affected by the advantage of a shorter cycle. In the genetic introgression hypothesis of *M. neotredecim* [34], alleles for the 13-year cycle are assumed to be strongly advantageous because 13-year life cycles seems to have some advantage in southern climates, while 17-year cycles survive better in the north [19]. However, our results suggest that advantage in climatic adaptation is not necessarily required for the replacement of 17-year life cycles by 13-year life cycles.

The most significant theoretical finding in our results is the key role that Allee effects [36] might play in life cycle evolution. Recently the importance of Allee effects as an extinction problem is recognized in empirical studies of ecology and conservation [43]. This is the second study suggesting the importance of Allee effects as an evolutionary factor. Allee effects have also been proposed as factors important in the selection of prime-numbered cycles [24], [28], [44], [45]. The uniqueness of our current model is that evolutionary outcomes depend on the relative strength of Allee effects; cycle shifts result from moderate to strong Allee effects, while brood formation results from weak Allee effects.
